# A clinician’s dilemma: what should be communicated to women with oncogenic genital HPV and their partners regarding the risk of oral viral transmission?

**DOI:** 10.1186/s12905-022-01965-x

**Published:** 2022-09-17

**Authors:** Ermelinda Monti, Giussy Barbara, Giada Libutti, Veronica Boero, Fabio Parazzini, Andrea Ciavattini, Giorgio Bogani, Lorenzo Pignataro, Beatrice Magni, Camilla Erminia Maria Merli, Paolo Vercellini

**Affiliations:** 1 Gynaecology Unit, Fondazione IRCCS Ca’ Granda Ospedale Maggiore Policlinico, Milan, Italy; 2 Regional Referral Center for the Prevention, Diagnosis and Treatment of HPV-Related Genital Disorders, Fondazione IRCCS Ca’ Granda Ospedale Maggiore Policlinico, Milan, Italy; 3 Department of Clinical Sciences and Community Health, Università degli Studi, Milan, Italy; 4 School of Midwifery, Università degli Studi, Milan, Italy; 5 Department of Odontostomatologic and Specialized Clinical Sciences, Università Politecnica delle Marche, Ancona, Italy; 6 Obstetric and Gynaecology Unit, Ospedali Riuniti Umberto I, Ancona, Italy; 7 Department of Maternal and Child Health and Urological Sciences, Sapienza University of Rome, Rome, Italy; 8 Otolaryngology – Head and Neck Surgery Unit, Fondazione IRCCS Ca’ Granda Ospedale Maggiore Policlinico, Milan, Italy; 9 Political Philosophy Unit, Department of Social and Political Sciences, Università degli Studi, Milan, Italy

**Keywords:** Human papilloma virus, Cervical cancer, Oropharyngeal cancer, Vaccination, Primary prevention, Counseling, Guideline

## Abstract

Head and neck cancer, the sixth most common cancer worldwide, account for about 1 out of 20 malignant tumors. In recent years a reduction in the incidence of cervical cancer, but a concomitant major increase in the incidence of HPV-mediated oropharyngeal cancer caused by orogenital HPV transmission has been observed. Consequently, in wealthy countries oropharyngeal squamous-cell carcinomas (OPSCC) is now the most frequent HPV-related cancer, having overtaken cervical cancer. Without effective medical interventions, this incidence trend could continue for decades. As no specific precursor lesion has been consistently identified in the oral cavity and oropharynx, HPV vaccination is the logical intervention to successfully counteract also the rising incidence of OPSCCs. However, HPV vaccine uptake remains suboptimal, particularly in males, the population at higher risk of OPSCC. Alternative primary prevention measures, such as modifications in sexual behaviors, could be implemented based on knowledge of individual genital HPV status. Until recently, this information was not available at a population level, but the current gradual shift from cytology (Pap test) to primary HPV testing for cervical cancer screening is revealing the presence of oncogenic viral genotypes in millions of women. In the past, health authorities and professional organizations have not consistently recommended modifications in sexual behaviors to be adopted when a persistent high-risk HPV cervicovaginal infection was identified. However, given the above changing epidemiologic scenario and the recent availability of an immense amount of novel information on genital HPV infection, it is unclear whether patient counseling should change. The right of future partners to be informed of the risk could also be considered. However, any modification of the provided counseling should be based also on the actual likelihood of a beneficial effect on the incidence of HPV-associated oropharyngeal cancers. The risk is on one side to induce unjustified anxiety and provide ineffective instructions, on the other side to miss the opportunity to limit the spread of oral HPV infections. Thus, major health authorities and international gynecologic scientific societies should issue or update specific recommendations, also with the aim of preventing inconsistent health care professionals’ behaviors.

## Introduction

Six malignancy types can be mediated by HPV, that is, cervical, vaginal, vulvar, anal, penile, and oropharyngeal cancer. In the United States (US) in 2014, 11,970 cervical, 860 vaginal, 2050 vulvar, 6460 anal, 860 penile, and 6830 oral plus oropharyngeal cancers were considered attributable to HPV [1]. In recent years a reduction in the incidence of cervical cancer, but a concomitant major increase in the incidence of HPV-mediated oropharyngeal cancer has been observed in wealthy countries. Orogenital HPV transmission has now overtaken smoke use and heavy alcohol consumption as the main risk factor for oropharyngeal cancers [2–6].

The gradual shift from cytology (Pap test) to high-risk HPV testing for cervical cancer screening is revealing the presence of oncogenic viral genotypes in a multitude of women. In an unvaccinated population, the proportion of participants with a positive test was as high as 27% at age 24–29 years, 13% at 30–39 years, 7% at 40–49, and about 5% at older ages [7].

Up to now, health authorities and professional organizations have not recommended modifications in sexual behaviors to be adopted in case of persistent high-risk HPV cervicovaginal infection, also because the risk of HPV-mediated penile cancer is very low [1, 8]. However, given the rapidly increasing number of HPV-mediated oropharyngeal cancers, it is unclear whether the type of counseling provided to the progressively increasing number of women with a persistently positive high-risk HPV test emerging from national cervical cancer screening programs should change. Are health care practitioners sufficiently aware and knowledgeable regarding not only cervical, vaginal, vulvar, anal, and penile, but also oropharyngeal HPV-mediated cancers? Should they address the issue of orogenital HPV transmission and, if yes, how?

Medical ethics also raises the question of how it is right to communicate within the patient-physician relation as well as in context of health campaigns and public interventions of medical experts.

Hypothetically, preventive measures could be recommended, modifications in sexual behaviors suggested, and information for future partners considered. However, any modification of the counseling usually provided to women with a positive cervicovaginal HPV test should be based not only on evidence, but also on the actual likelihood of a beneficial effect on the incidence of HPV-associated oropharyngeal cancers, as well as on the potential harms associated with novel recommendations. The risk is, on one side to induce unjustified anxiety and provide ineffective instructions, on the other side to miss the opportunity to limit the spread of oral HPV infections.

After a brief overview of epidemiological and pathogenic data, whether addressing the issue of orogenital HPV transmission in women with a persistent genital HPV infection appears reasonable will be here considered. This article is not meant to be exhaustive nor to examine all the aspects potentially related to this matter. Which type of information, if any, should be added to patient’s counseling is the exclusive duty of health authorities and professional organizations.

## Epidemiological background of oropharyngeal HPV infection

Head and neck cancer, the sixth most common cancer worldwide, account for about 1 out of 20 malignant tumors [9]. Around two thirds of head and neck cancers are identified at late stages [9]. Squamous cell carcinomas occurring in the oral cavity, oropharynx, hypopharynx, and larynx mucosae constitutes over 90% of head and neck cancers [10]. Specifically, oropharyngeal squamous cell carcinomas (OPSCCs) arise in the epithelium of the crypts of the palatine and lingual tonsils, base of tongue, soft palate, and posterior wall of pharynx [10].

Since the 1970s, an increase in the incidence of mucosal squamous cell carcinomas of the head and neck, and particularly of OPSCCs, has been consistently observed, despite a decrease in the exposure to smoking and heavy alcohol consumption. Indeed, from 1990 to 2017, incidence rates for nasopharyngeal, laryngeal, and lung cancers decreased worldwide [4, 5]. This variation appears to be related to the rising incidence of HPV infection of the oropharyngeal mucosa transmitted from anogenital sites secondarily to increased orogenital sexual activities and number of sexual partners [3, 5]. The number of oral sexual partners is the risk factor for OPSCCs most consistently identified [4].

Mainly because of effective screening, the incidence of cervical cancer in the western society is decreasing (7.4 per 100,000). As over 70% of OPSCCs are attributable to HPV, in the US they are now the most frequent HPV-related cancer, having overtaken cervical cancer [11]. It has been estimated that by 2030 the majority of all mucosal squamous cell carcinomas of the head and neck will be HPV-related [5].

Without effective medical interventions, this incidence trend could continue for decades [12]. According to Roman and Aragones [13], by 2030 the incidence of OPSCCs in White men aged 65–74 is expected to rise to more than 70 in 100,000, whereas in White women of the same strata of age the increase is estimated to plateau to about 10 in 100,000. Moreover, the incidence of HPV-related OPSCCS is much higher than either at the anus or penis [14].

Such a sharp increase in HPV-related OPSCCs mainly affects men (7.8 per 100,000) [4, 5, 15]. In the decade 2002–2012, the incidence of OPSCCs among women remained substantially stable, whereas it increased dramatically among men (2.9% per year) [15].

Although the natural history of the transition to oropharyngeal cancer is not fully clarified, it is now commonly accepted that a subclinical oral HPV infection persisting for 10–30 years is an obligate precursor of the majority of OPSCCs [12, 13, 16] Indeed, HPV detection in OPSCCs increased dramatically over time and several high-risk types have been identified, including 16 and 18, 31, 33, 35, 52, 58 (all genetically related to HPV 16); and 39, 45, 59 (genetically related to HPV 18), with HPV 16 accounting for 70–80% of cases [17, 18].

In the large National Health and Nutrition Examination Survey, the overall oral HPV infection prevalence was 11.5% in men and 3.2% in women. Prevalence estimates for high-risk HPV oral infection was 7.3% among men and 1.4% in women. In particular, the prevalence of oral HPV 16, the genotype associated with over two-thirds of OPSCCs, was 1.8% and 0.3%, respectively. The prevalence of high-risk oral HPV was 13.7% in men with concomitant genital infection and 3.9% in those without. Corresponding figures in women were 3.2% and 1.1% [15].

The overall oral HPV clearance rate appears highly variable between studies, with a median time to clearance from 6 to 18 months [12]. For oral HPV 16, the clearance rate was 43–83% and the median time to clearance was 7–22 months [11, 12]. In the HPV Infection in Men Study, median time to viral clearance was around six months, whereas persistence of high- and low-risk HPV was approximately 20% and 3%, respectively, with percentages varying from 31.8% for HPV 39 to 18% for HPV 16. The study population was composed of men who have sex with men only (MSM), men who have sex with women only (MSW), and men who have sex with women and men (MSW). Overall, 10–30% of these infections persisted at two-year follow-up [16].

## Dynamics of oropharyngeal HPV transmission

Oropharyngeal HPV infection occurs through contact between the mouth and anogenital region, whereas few infections would be caused by open mouth kissing [13, 19]. The bidirectional transmission of HPV between the genital and the oral area acts as a promoter of OPSCCs in both women and men [15]. However, the reason why the risk of persistent oral HPV infection is so much higher in men than in women remains unexplained, as the prevalence of genital HPV infection is comparable in both sexes [2]. Investigations on individual sexual behaviors are subjected to different information bias that may limit the reliability of associations between number of partners, timeframes, frequency and order of orifice exposure, as well as possibly adopted preventive measures [20].

It has been hypothesized that men have more oral sexual partners than women, therefore, more chance of oral exposure to HPV [5]. However, when analyzing 2011 to 2015 data from the National Survey of Family Growth, the proportion of individuals giving and receiving oral sex was similar among women and men [21]. Therefore, additional factors might play a role, and disentangling this issue could have potential preventive implications.

Some investigators suggest that performing oral sex on female genitalia (i.e., a mucosal surface) leads to a higher transmission rate of infection as compared to the transmission occurred when performing oral sex on male genitalia, because of the keratinized epithelium of the penis. In addition, it has been suggested that the keratinized epithelium of the penis is more resistant to HPV infection than the epithelium of the cervix [14]. Whether the amount of biological fluid reaching the oral cavity might also determine contagiousness has not been studied. 

Finally, women may develop a stronger systemic immune response than men after genital HPV infection. This would more efficiently protect women compared with men in case of subsequent oral exposure to HPV [4, 14]. This hypothesis seems to be supported by the observation that, among men and women who reported having same-sex partners, the prevalence of high-risk oral HPV infection was 12.7% and 3.6%, respectively, whereas among men and women who did not report this behavior, it was 6.8% and 1.2%. Moreover, among men who reported having 2 or more same-sex oral sex partners, the prevalence of high-risk HPV infection was 22.2% [15]. Therefore, the risk of oral infection in men is not necessarily associated only with giving oral sex to women, but also to men [21].

Other modalities of oral HPV contamination have been hypothesized, including autoinoculation and partner infection by means of transmission between genitals, anal canal, hands, and oral cavity [14, 15]. Oral HPV DNA was identified in up to one fifth of women with a diagnosis of cervical-HPV and almost one third of their male partners [22], but correspondence between cervicovaginal and partner oropharyngeal HPV types is limited. Even concordance of oral-genital HPV genotypes in the same woman and man is inconsistent [2, 15]. This impedes oversimplifications on the origin of the oral infection. Nonetheless, male partners of women with cervical cancers had substantially increased risk of developing HPV-mediated tongue and tonsil cancer [23].

## Screening, early diagnosis, and prevention of oropharyngeal cancers

The very long incubation period between oral infection and cancer development complicates strategies aimed at early diagnosis of HPV-related OPSCCs. More importantly, no specific precursor lesion has been consistently identified in the oral cavity and oropharynx [9, 12, 24, 25]. Most HPV-related neoplasia originates in the epithelium lining the deepest portion of tonsillar crypts. Thus, the reliability of the examination of the oropharyngeal mucosa is limited by the fact that precisely those early lesions that would be the object of screening are generally concealed from visual inspection [22, 24, 25]. For the same reason, a cytology screening (scrapings or brushing of the oropharyngeal mucosa) may miss precancers located deeply in the tonsillar crypts [12].

A molecular assay for high-risk HPV DNA has been considered an alternative to oral Pap test [22]. To this aim, saline mouthwash seems to perform better than swabs from the tonsillar fossa [12, 26]. However, oral HPV screening would detect the presence of HPV DNA in the saliva, thus identifying an active infection, but not necessarily a pre-malignant lesion, as most infections would be cleared or, if persistent, would not lead to malignant derailment [24, 25]. Even when a persistent infection with an oncogenic HPV type is demonstrated at repeated oral HPV-DNA testing, in most cases a precursor lesion would be difficult to identify. Thus, contrarily to cervical cancer screening, where HPV testing allows successful cytologic and histologic detection of precancerous lesions in women with high-risk viral subtypes, the conditions for effective and cost-effective oral HPV screening of the general population are not met.

A dedicated follow-up program could be targeted toward specific high-risk subpopulations, e.g., men aged 50–65 years reporting previous multiple oral sexual partners or individuals with persistent oral HPV 16 infection [9, 15, 24, 25]. However, no sufficient data is available to quantify the potential effect of such programs. Indeed, the US Centers for Disease Control and Prevention (CDC) recommend against performing oral HPV testing in the general population as well as in women with genital HPV infection and their partners [27].

Until uncertainties on screening and early diagnosis are disentangled, primary prevention remains the only mean to successfully counteract the rising incidence of OPSCCs [11, 28]. The currently available 9-valent vaccine appears to protect against infection with viral subtypes associated with over 90% and almost 80% of HPV-related cancers of the oropharynx and larynx, respectively [4]. In June 2020 the US FDA extended the indications for the 9-valent HPV vaccine to include the prevention of oropharyngeal cancers in both sexes [29] However, the uptake of HPV vaccination in boys remains worryingly low [11], despite the US CDC foster vaccination of females and males starting from 9 years of age [30]. Moreover, US CDC suggests that for women and men aged 27–45 years, the benefit of HPV vaccination in terms of improvement in public health should be considered minimal, and that in this specific situation a shared clinical decision-making is strongly recommended [31].

The Society of Obstetrics and Gynaecology of Canada (SOGC) has recently published a statement on HPV-associated cancers summarizing recommendations on vaccination, screening, and early detection [28]. According to this document, the HPV vaccination rate remains low due to lack of doctors and citizens awareness of HPV infections and HPV-mediated cancers prevalence and vaccine efficacy. The only measure fostered by the SOGC to prevent HPV-associated head and neck cancers is vaccination for all individuals aged 9–45 years, or any age with on-going risk of exposure to HPV. Screening and early diagnosis are deemed practically unfeasible for this cancer category [28]. However, the absence of established precancerous oropharyngeal lesions to assess the HPV vaccine’s effectiveness could represent an obstacle, as also does physician’s poor awareness regarding extra-genital HPV driven neoplasia*.*

Population attitude toward HPV vaccination is largely influenced by physicians’ recommendations. Not encouraging vaccination whenever possible constitutes a missed clinical opportunity [32]. The potential for primary prevention of HPV-related head and neck cancers in both women and men should be emphasized, as it may not be excluded that reluctance to vaccinate boys may be partly based on the conviction that the benefit would be a decrease in the incidence of cervical cancer only, and that this goal could be achieved anyway vaccinating girls. Of note, men are substantially less informed than women regarding the associations between HPV, cancer, and vaccination, despite three fourth of OPSCCs occur in men [33].

The HPV vaccination should be recommended by all health care practitioners, including oral health care specialists, otolaryngologists, family physicians, nurse practitioners, nurse clinicians, and pediatricians, as they could represent a significant source of information for the enrollment of un-vaccinated individuals [34].

Smartphone applications have been developed to promote behavior change regarding HPV vaccination in physicians [32, 35]. These digital programs seem easy to use and effective in increasing awareness and knowledge on modalities of HPV spread and possibilities of prevention. Apps and telemedicine could be explored as potentially successful means, in addition to standard campaigns, also to reach the adolescent population.

As oro-genital sex is the risk factor most associated with HPV-related OPSCCs, the only alternative primary prevention measure that could influence the incidence of oral HPV infections is the adoption of barriers, that is, condoms and dental dams, to limit the exchange of body fluids or contact between mucous membranes [24, 25]. The use of condoms and dental dams to cover the genital and anal areas is suggested by the CDC [36]. However, as barriers may not cover entirely the areas of infected skin, viral transmission may occur also via skin-to-skin and skin-oral contacts.

Moreover, whereas the use of condoms at vaginal and anal intercourse is very common and largely accepted by both partners, the uptake of barriers at orogenital sex appears much more limited. The regular use of condom at oral sex has been observed to have remained stable during the past decade at a rate as low as 6–7%. Corresponding data for dental dam use are scanty and, overall, indicate even lower adoption rates [15, 21, 37]. A study on misperceptions regarding protective barrier method use for safer sex among women who have sex with women (WSW) reveals that dental dams and female condoms are rarely used by WSW [38]. It has been reported that among Australian women who had had oral sex with a woman in the previous 6 months, 9.7% had used a dental dam and 2.1% had used one “often” [39]. Beyond the currently highlighted benefit on the spread of sexually transmitted infections, stronger emphasis should be given to the potential effect of barrier use at orogenital sex specifically in terms of potential reduction in the risk of HPV-related head and neck cancers [24, 25, 38],

The American College of Obstetricians and Gynecologists includes household plastic wrap among barrier protections to be used when women receive oral sex, tough acknowledging that no data on its effectiveness are available and that U.S. Food and Drug Administration has not evaluated or cleared it for this specific use [37]. Plastic films can easily protect a larger perineal area than dental dams, interfering slightly with tactile and visual stimuli. Assessment of whether common domestic polyethylene food coverings successfully reduce the likelihood of HPV transmission from the vulvar and anal areas to the oral cavity would be useful, in the hypothesis that their acceptance, especially by younger couples, could be higher than that of thicker and more expensive dental dams. If proven effective and tolerated, the use of polyethylene food films with future partners could be suggested to women with persistent high-risk HPV cervicovaginal infection who are reluctant to adopt dental dams.

## Informing women with persistent high-risk HPV genital infection: practical and bioethical issues

Orogenital sex is practiced by about half of adolescents and almost all adults, but awareness of the risks associated with receiving and giving oral sex is limited and emphasis is generally put on infection via vaginal intercourses [15, 21, 37]. Moreover, oral sex is not at risk of unwanted pregnancies and the motivation toward the use of barriers may be weaker than at vaginal sex [37]. However, transmission of an oncogenic HPV to the oropharynx appears substantially riskier than to the penis, as the incidence of HPV-related OPSCC, often diagnosed at late stages with lymph node involvement, is manyfold higher than that of HPV-related penile cancer [6, 8].

The US CDC suggest informing women with positive HPV-DNA test that sex partners also are likely to have an HPV infection but do not need to be tested, considering that the benefit of revealing a positive HPV condition to current and future sexual partners is doubtful. Indeed, according to the CDC recommendations, HPV infection should not have to raise concerns about a male partner’s health” [27]. Therefore, at present, oral HPV testing and follow-up of partners of women with a persistent oncogenic HPV genital infection may not be suggested outside approved clinical studies. Moreover, no change in usual sexual practice is required with current or long-term partners [19].

When addressing health risks of noncoital sexual activity, the American College of Obstetricians and Gynecologists synthetically state that oral HPV transmission can occur and that performing oral sex is associated with the risk of oropharyngeal cancer, and suggest encouraging women to use barrier protection during oral sex, although acknowledging that most individuals are unlikely to use it [37].

Information for lay people by UK NHS regarding orogenital sex includes explanation on the risk of transmission of “genital warts”, but not of high-risk oral HPV infection. Moreover, the use of condom is recommended, but that of dental dams is not addressed [40].

In the recently update CDC Sexually Transmitted Infections Treatment Guidelines, 2021, only consistent and correct condom use is recommended as a barrier method to reduce the risk of transmitting HPV infection, although the possibility of viral spread from the anogenital area to the mouth and throat, as well as development of head and neck cancers in case of persisting HPV infection, are indicated [27]. Instruction from CDC on how to use dental dams are available at a different site [41]. Emphasis is put on barriers as a measure to reduce the risk of genital warts and cervical cancer, but no mention is made of oropharyngeal cancer [42].

According to information provided to patients with HPV-related OPSCCs by the Johns Hopkins Hospital, no modification in sexual behavior with the current partner is needed, as likely the infection has been already shared. However, the use of condoms and barrier protection should be discussed with new sexual partners in the future [43].

Currently, the information process on the potential harms of orogenital sex in women with persistent high-risk HPV genital infections appears problematic. Firstly, the knowledge of gynecologists regarding the increased, although limited, risk of oropharyngeal cancer in both the woman and her prospective partners is generally limited. Secondly, even if the increase in the individual absolute risk is small, whether future partner(s) should be informed about the condition is unclear. The evidence on which to base counseling is still undefined, and the US CDC does not encourage this disclosure. However, prospective partners may have the right to know the potential consequences of being contaminated with an oncogenic HPV when giving unprotected oral sex to women with persistent genital HPV infections (in particular in women with persistent high-risk HPV infection, as the vast majority of oropharyngeal cancers are HPV16-driven), independently of the magnitude of the increase in risk. Thirdly, the issue of whether women who prefer not to inform their future partners and at the same time refuse to use dental dams, should reveal their condition before receiving oral sex without barriers has not been adequately addressed. The same issues also apply in the opposite direction in the case of men who test positive at a penile HPV-DNA test. However, no screening is in place to detect penile HPV. Moreover, uncertainties regarding viral latency and the ability of individuals harboring HPV active infections below the DNA positivity threshold to contaminate their sexual partners could pose additional challenges in patient counseling [44].

To date, evidence-based answers to these issues seems lacking and this could generate two types of problems. On the one hand, the potential harms could be overemphasized, thus causing undue psychological distress and the adoption of excessively protective but not necessarily effective behaviors. On the other hand, those risks could be underestimated or dismissed, and this could result in a further increase in the incidence of partly preventable oropharyngeal cancers.

For now, women with persistent HPV genital infections and their partners should be instructed to avoid or cease smoke and heavy alcohol consumption, as these are strong co-factors that can favor progression of HPV-mediated oropharyngeal cancers [3, 6].

The above considerations on epidemiological and pathogenic data also raise the issue of the ethics of health communication. Patient-physician communication has ethical relevance because it constitutes time of care, is necessary for the clarification of values and preferences, and can change the patient's reality. Many of the actions that the doctor undertakes every day involve ethical issues, often implicit, but which become evident in the presence of a conflict of values. Of these actions, a good part involves communication, directly or indirectly. An example of action involving communication in the context of the patient-physician relationship is, as in this case, the information about risks. According to any sense of autonomy, the autonomous subject is the one who manifests personal values and preferences. Indeed, there is no autonomy without expression of autonomy and, therefore, without communication. Therefore, in an ethical framework in which there is a role for autonomy as a principle, there must be consideration for bidirectional communication [45].

## Conclusion

Educating the population to accept systematic vaccination of girls and boys may take much time. Awareness of, interest in, and knowledge on OPSCC might all increase if HPV-mediated oropharyngeal cancer is included by health authorities and professional organizations among sexually transmitted diseases. Such a definition would stimulate physicians to foster gender-neutral vaccination of the adolescent population, whose uptake is still well below the optimal target especially in males.

However, given the very long latency period between oral HPV infection and OPSCC development, data on the actual magnitude of the effect of vaccines in the prevention of OPSCCs would be available in some decades. Differently from cervical cancer, no precursor lesions can be currently investigated as surrogate markers to allow early evaluation of vaccine effectiveness [46].

During this time, health authorities and professional societies may decide not to use the newly available tens-of-millions positive HPV tests deriving from recent national cervical cancer screening programs outside the primary objective or, alternatively, hypothesize the use of this immense information for trying to reduce also oropharyngeal cancer incidence through counseling and recommendations targeted at women with a persistent oncogenic HPV genital infection.

On one hand, the question is what would be the value of suggesting modifications in orogenital sex practices with future partners, including the adoption of barriers, to this subpopulation? Such recommendations may imply potential harms, i.e., adverse emotional responses, anxiety, reduction of self-esteem, stigma, embarrassment, interference with intimacy, sexual distress, need for explanation and consequent partner mistrust [19, 47]. Even the mere positivity of a HPV test, often leads to stigmatization of the woman and blaming of the female sexual partner, or causes detrimental effects in the emotional aspects of sexual relationships. Moreover, the degree of understanding of the meaning of an HPV test result and of the related practical implications may be scarcely predictable, as successful communication is influenced also by individual education levels [48].

The potential benefits seem realistic, but are quantitatively undefined and possibly small, as partners may have been already infected in the past or could be infected in the future by other unaware carriers of genital HPV. In addition, orogenital sex is the main, but not the only modality for transmission of HPV in the oral cavity. Finally, HPV is the most important, but not the only factor determining the development of OPSCCs.

On the other hand, the right of prospective partners to be informed by women who are cognizant of their status of genital HPV carrier could also be considered. No HPV screening is suggested in men. Therefore, although HPV transmission is bidirectional, this ethical issue arises typically when women became aware of their oncogenic HPV genital infection. In this case quantification of the benefit deriving from communication seems less decisive, as different partners may accept different levels of risk. One may be willing to be informed anyway and avoid the risk even if minimal.

Finally, data on the effectiveness of dental dams and plastic films for the reduction of orogenital HPV transmission are still insufficient. The potential benefit is also here undefined, whereas the potential harms include embarrassment and reduced pleasure. Obviously, avoiding sexual intercourses is not acceptable.

Hopefully, ongoing studies will disentangle some of these uncertainties, but again this will take time [49]. Prevalence, incidence, and persistence of oral HPV in women and men, as well as the factors associated with oral infection, will be assessed in the PROGRESS study [50], whereas the main objective of the BROADEN study is to define the proportion of head and neck cancers attributable to HPV by anatomic site. [51]. The efficacy of the 9 valent HPV vaccine against persistent oral HPV in men will be evaluated in a phase 3, multicenter, randomized, double-blind, placebo-controlled trial in which 6000 participants will be recruited [52, 53].

Meanwhile, more detailed recommendations on how women with a persistent high-risk HPV-DNA test should be counseled regarding orogenital sex and the risk of oropharyngeal cancer should be issued by major health authorities and international gynecologic scientific societies. Communicating uncertainties would be also important but one thing is communicating “official uncertainties”, another thing is communicating personal uncertainties [54, 55]. Even in case no change in the currently approved counseling for this subpopulation is deemed necessary in light of the rising OPSCC incidence, updated explicit statements including explanations would be appropriate and would prevent inconsistent gynecologists’ behaviors.

In general, many of the specific questions that arise in the field of patient-physician communication ethics concern the balance between paternalism and autonomy. There are here several ethically complex dimensions that gynaecologists can face when caring for these women, including respect for autonomy, confidentiality, epistemic justice, sincerity, and consent. In each of them there is no single way out, but the ethics of communication requires reasoning about the context of the matter and principles. The decision-making autonomy of the patient is achieved only with adequate knowledge of everything related to one's condition, and this knowledge can only derive from a successful communicative exchange in both directions between patients and physicians [56].

What to do then? Each health promotion strategy reflects not only a certain concept of health and well-being, but also a precise idea of what an equitable society is and what the priority values are. Respect for autonomy, however conceived, within an empowerment strategy, must not prevail over the principle of charity, or the ideal of the public good, which is the basis of health promotion interventions. Empowerment, in other words, must never become for health institutions an intrinsic end to be pursued, but always a mean to improve people's health and well-being [57].

## Data Availability

Not applicable.

## Abbreviations

HPV: Human papilloma virus
OPSCC: Oropharyngeal squamous-cell carcinomas
US: United States
CDC: Centers for disease control and prevention
SOGC: Society of obstetrics and gynaecology of Canada
ICMJE: International committee of medical journal editors
